# Supplementary material for: Odorant Binding Proteins (OBPs) and Odorant Receptors (ORs) of Anopheles stephensi: Identification and comparative insights
Source: PLoS One. 2022 Mar 22;17(3):e0265896. doi: 10.1371/journal.pone.0265896 (PMC8939812; doi:10.1371/journal.pone.0265896)
Supplement: S1 Table — ORFs were predicted by ORF Finder. Similarly, OBP’s names and accessions have also been given. (DOCX) [file pone.0265896.s005.docx]

Supplementary Table 1. Nucleotide length of the open reading frame (ORF) and protein length in the OBPs have been provided along with their complete and partial status. ORFs were predicted by ORF Finder. Similarly, OBP’s names and accessions have also been given.

| OBP Name | Protein Accession | ORF | ORF Length | Protein Length |
| --- | --- | --- | --- | --- |
| AsteOBP1 | XP_035917841.1 | Complete | 507 | 168 |
| AsteOBP2 | XP_035891203.1 | Complete | 438 | 145 |
| AsteOBP3 | XP_035891204.1 | Complete | 435 | 144 |
| AsteOBP4 | XP_035891207.1 | Complete | 438 | 145 |
| AsteOBP5 | XP_035891740.1 | Complete | 423 | 140 |
| AsteOBP6 | XP_035891741.1 | Complete | 405 | 134 |
| AsteOBP7 | XP_035892015.1 | Complete | 411 | 136 |
| AsteOBP8 | XP_035892546.1 | Complete | 1377 | 458 |
| AsteOBP9 | XP_035892547.1 | Complete | 1041 | 346 |
| AsteOBP10 | XP_035893080.1 | Complete | 486 | 161 |
| AsteOBP11 | XP_035894183.1 | Complete | 615 | 204 |
| AsteOBP12 | XP_035894335.1 | Complete | 429 | 142 |
| AsteOBP13 | XP_035894381.1 | Complete | 441 | 146 |
| AsteOBP14 | XP_035895118.1 | Complete | 840 | 279 |
| AsteOBP15 | XP_035895684.1 | Complete | 411 | 136 |
| AsteOBP16 | XP_035895818.1 | Complete | 480 | 159 |
| AsteOBP17 | XP_035895821.1 | Complete | 405 | 134 |
| AsteOBP18 | XP_035897326.1 | Complete | 738 | 245 |
| AsteOBP19 | XP_035897725.1 | Complete | 441 | 146 |
| AsteOBP20 | XP_035898485.1 | Complete | 441 | 146 |
| AsteOBP21 | XP_035898834.1 | Complete | 546 | 181 |
| AsteOBP22 | XP_035898836.1 | Complete | 450 | 149 |
| AsteOBP23 | XP_035899548.1 | Complete | 456 | 151 |
| AsteOBP24 | XP_035900970.1 | Complete | 396 | 131 |
| AsteOBP25 | XP_035902367.1 | Complete | 441 | 146 |
| AsteOBP26 | XP_035903187.1 | Complete | 408 | 135 |
| AsteOBP27 | XP_035903789.1 | Complete | 507 | 168 |
| AsteOBP28 | XP_035905778.1 | Complete | 480 | 159 |
| AsteOBP29 | XP_035907589.1 | Complete | 606 | 201 |
| AsteOBP30 | XP_035907591.1 | Complete | 588 | 195 |
| AsteOBP31 | XP_035907600.1 | Complete | 612 | 203 |
| AsteOBP32 | XP_035908250.1 | Complete | 630 | 209 |
| AsteOBP33 | XP_035908252.1 | Complete | 537 | 178 |
| AsteOBP34 | XP_035908253.1 | Complete | 528 | 175 |
| AsteOBP35 | XP_035909003.1 | Complete | 969 | 322 |
| AsteOBP36 | XP_035910841.1 | Complete | 465 | 154 |
| AsteOBP37 | XP_035914753.1 | Complete | 834 | 277 |
| AsteOBP38 | XP_035917598.1 | Complete | 420 | 139 |
| AsteOBP39 | XP_035895071.1 | Complete | 537 | 178 |
| AsteOBP40 | XP_035895083.1 | Complete | 420 | 139 |
| AsteOBP41 | XP_035907843.1 | Complete | 963 | 320 |
| AsteOBP42 | XP_035907857.1 | Complete | 891 | 296 |
| AsteOBP43 | XP_035907868.1 | Complete | 882 | 293 |
| AsteOBP44 | XP_035907882.1 | Complete | 834 | 277 |
