# Supplementary material for: Odorant Binding Proteins (OBPs) and Odorant Receptors (ORs) of Anopheles stephensi: Identification and comparative insights
Source: PLoS One. 2022 Mar 22;17(3):e0265896. doi: 10.1371/journal.pone.0265896 (PMC8939812; doi:10.1371/journal.pone.0265896)
Supplement: Attachment — Submitted filename: Response to Reviewer 2.docx [file pone.0265896.s007.docx]

Response to Reviewer 2

**Point 1.** Considering that this is a major malaria vector in some parts of the world, it is surprising that there has not been a comprehensive report on olfactory genes in this species, as has been done for other mosquito disease vectors. So this manuscript would most certainly be worthy of publication, however major revisions to the text and figures are required before this manuscript would be suitable for publication. Many of the figures are low resolution. I am uncertain if this is a consequence of assembly of the images in a PDF, or of low-resolution images were initially provided by the authors, but it is difficult to fully assess especially the OR phylogeny relative to some of the claims made by the authors, so a better resolution image would be needed, as it is now, the text in this figure is too blurry to read.

**Response 1.** Thank you for your kind response. Figures are in very good resolution. Their resolution has been distorted during the assembly of pdf. These can be downloaded and are easier to read and understand. Figure of ORs phylogeny has been improved to make in understandable and readable.

**Point 2.** The manuscript will also need to be thoroughly edited for language after all revisions have been made. There are numerous errors throughout the text. Furthermore, the language is not always clear nor well-structured. Careful revisions with respect to grammar, structure/organization and word choice needs be considered.

**Response 2.** The manuscript has been revised for the grammatical errors and unclarity in certain sentences. To the best of knowledge, grammatical errors have been removed from the text and sentence structure has been improved.

**Point 3.** Description in the methodology is not always clear. Specifically, in the first section “Identification of the OBPs and ORs”, it is mentioned that proteome fasta files are examined for OBPs and ORs (Lines 96 and 114). Then on Lines 110-112 it is mentioned.

-OBPs were renamed according to their position on the chromosomes. Chromosomal positions of the OBP encoding genes were visualized…-

**Response 3.** Thank you for pointing out. Word proteome has been replaced by protein as it is pertinent to mention protein FASTA file. Similarly, the manuscript including methodology has been improved. Then line 110-112 has been modified in the manuscript.

**Point 4.** However, it has never been made clear in the manuscript that an *An. stephensi* genome has been examined. There is no citation or reference to a genome for this species anywhere, and it is clear that the authors have conducted some studies of these genes in the genome, so this basic information on the source of the genome would have to be clearly indicated in the appropriate section(s). This is also apparent in that in the supplemental materials file, in which nucleotide as well as protein sequences are presented for the ORs and OBPs of An. stephensi, however it is not at all clear where the nucleotide sequences have been derived from, considering that the authors only mention about proteome fasta files, and it is not clear where these proteome files are derived from. It is not always the case that proteomes are directly connected to sequenced genomes.

**Response 4.** Genome assembly of *An. stephensi* Indian strain (GenBank assembly accession. GCA_013141755.1) was used for the analysis. It has been modified in the manuscript along with the citation of the genome.

**Point 5.** The authors mention in the introduction about the odorant receptor co-receptor (Orco), but it is not clear in Table 2 nor any of the figures dealing with the ORs (Fig 3, 6, 8 and 10) that they are reporting on the Orco orthologue for this species. Has this critically important gene been omitted in this report?

**Response 5.** Thanks for pointing out. This study is focused on the ORs and OBPs. ORco has not been incorporated into the study.

**Point 6.** There are no statistical measurements provided for phylogenetic relationships in Figures 5 and 6, such as bootstrap values of maximum likelihood support values. These values are essential to ensure strength of relationship between different genes and nodes in the tree.

**Response 6.** Thanks for pointing out. Bootstraps values have been in both phylogenetic trees and updated in the manuscript as well.

**Point 7.** Table 1 and Table 2 both show basic information on the OBPs and ORs identified in An. stephensi, however this information as shown is not critically useful, especially the Protein Accession and Gene ID, which is more suitable to be included as a supplemental info. For a main-text table, such as these, it would be better to include other metrics such as completion status of the open reading frame (complete/incomplete) as well as the size of the protein (number of amino acids, molecular weight) and also isoelectric values. It is also common to show the geneID of the best blast hit from other species for each novel gene identified.

**Response 7.** Thank you for suggestion. Modifications have been made in the table as suggested. Protein size, molecular weight and isoelectric points have been provided. Syntenic analyses were performed to compare the genes of *Anopheles stephensi* with *Anopheles gambiae,* and *Aedes aegypti* for homology between these organisms.

**Point 8.** In fact, the isoelectric values and molecular weight values are summarized in Figure 11, but the actual result metrics are not presented anywhere in the report. At the very least, these values should be included as supplemental info, but could also be incorporated into a revised version of Tables 1 and 2.

**Response 8.** These values have been added in the revised table as per your kind suggestion. Thanks for the suggestion.

**Point 9.** With respect to the Isoelectric and Molecular Weight values, are only ORs and OBPs with complete open reading frames being considered? Because those are the only ones which could usefully be compared. It would be essential to clarify on this in the appropriate Materials and Methods section.

**Response 9.** Yes, only those OBPs having complete ORF were considered. This has been clarified in the manuscript.

**Point 10.** Line 26. “from protein sequences of the An. stephensi” Again here it is not clear what is the source of these protein sequences. This needs to be clarified according to my previous comment, throughout the text and at the appropriate sections.

**Response 10.** This has been clarified through the manuscript.

**Point 12.** Line 35. “whereas only three OBPs and two ORs were related to the Ae. aegypti OBPs and ORs.”

Are you sure about that? Aside from difficulties in clearly reading the phylogenetic trees due to low figure resolution, it is not accurate to suggest that genes are related only if they are directly next to each other with most recent common ancestry on the phylogenetic tree. All genes within a subfamily may indeed be related to each other provided statistical support for the clustering, even if they are not the most closely related genes within the cluster.

Several times throughout the text there is statements about relatedness or lack of relatedness. Please ensure clarifications or corrections are made to these kinds of statements throughout the entirety of the manuscript.

**Response 12.** These statements have been modified throughout the manuscript as per your suggestion. Thank you for pointing out.

**Point 13.** Line 72. “In An. stephensi, two OBPs have been identified but no ORs as of yet.”

This needs to be updated, as a publication this year, Speth et al., 2021, Insects, has reported on the characterization of an OR in An. stephensi.

**Response 13.** Thank you for suggestion. This has been updated in the manuscript.

**Point 14.** Line 76-77 “ORco” should be written as “Orco” according to convention established by Vosshall and Hansson, 2011, Chemical Senses.

**Response 14.** It has been modified in the manuscript.

**Point 15.** Line 133. “between the chromosomes of the two genomes”.

Which two genomes? In the appropriate methods section, it should be clearly written out which versions of which genomes are being analyzed for each species, and some kind of source information (accession number, literature reference, etc.) for each genome, in order to provide clear indication of which datasets are being analyzed.

**Response 15.** Between the chromosomes of the two genomes means that MCScanX predicts syntenic relationship between two different genomes. Source information has been added in the manuscript for the genomes that were used for comparison.

**Point 16.** Line 151. “were predicted were predicted” needs to be fixed.

**Response 16.** This line has been fixed.

**Point 17.** Line 169. “Renaming of ORs and chromosome”.

What is meant here by “renaming”? This is apparently the first description of ORs in this species, so how is it they are being renamed? Should say something like “Description or Classification of ORs” as is written for Table 1 on Line 161.

**Response 17.** As per your kind suggestion, word “renaming” has been omitted and replaced by naming. As OBPs and ORs were named as AsteOBPx and AsteORx as a convention used by Zhou, J‐J., 2008.

**Point 18.** Line 183/Line193. “Chromosome 2 is saturated with…”

This is incorrect scientific terminology. “Saturated” has a very specific meaning in chemistry and its usage here is most certainly incorrect, in that the chromosomes probably could contain more OR/OBP genes than they already have. In this sense, they would not be saturated. Please consider another word choice for these descriptions.

**Response 18.** As per your suggestion, word “saturated” has been omitted and sentence has been restructured.

**Point 19.** Line 253-254. “None of the An. stephensi OBPs and ORs shared homologous regions with the OBPs and ORs of D. melanogaster.”

It is not clear what this is referring to. Homologous regions of the protein sequence? Or referring to genomic synteny? This should be more clearly written to accurately describe what is being compared.

**Response 19.** Thanks for pointing out. This was referred to the genomic synteny. Such sentences have been corrected to bring more clarity.

**Point 20.** Line 260. “Contained insignificant domains”.

What is an insignificant domain? If such descriptions are going to be made, they need to be clearly defined in the appropriate Materials and Methods section.

**Response 20.** Insignificant domains mean that Plus-C OBPs had insignificant matches to the Pfam PBP/GOBP domain family. But these sequences were also analyzed using the multiple sequence alignment. Presence of conserved cysteine residues as reported by Zhou, J‐J., 2008 indicated these are plus-C OBPs. Therefore, these have been included in the study. This has been clarified in the manuscript.

**Point 21.** Line 287. “CELLO and WoLF PSORT predicted…” In the materials and methods section these softwares are mentioned but it is not clear how these determinations have been made. It would be more useful to indicate that identified OBPs contain Signal Peptides and that ORs contain appropriate number of transmembrane domains. There are specific softwares that analyze these features, but if that is what the indicated softwares have done, it would be important to clarify this in the Materials and Methods section.

**Response 21.** Protein sequences of the OBPs and ORs were used in the CELLO and WoLF PSORT webserver to predict the subcellular localization. Both servers are efficient in predicting the subcellular localization of OBPs and ORs. This has been added in the manuscript as per your suggestion. As this study is based on the identification of OBPs and ORs whereas signal peptide and transmembrane regions have not been predicted.

**Point 22.** Line 291-292. “OBPs and ORs are expressed at very high level in insects”.
This is not necessarily correct. ORs are not generally expressed at very high levels (with sometimes exceptions for Orco and sometimes pheromone receptors), and Some OBPs are indeed expressed at very high levels, but many of them are not. This needs to be corrected.

**Response 22.** Thanks for pointing. This line has been removed from the manuscript as several OBPs and ORs have low expression.

**Point 23.** Line 301 and 302. “Aenasius bambawalei” and “Tomicus yunnanensis”.

What kind of species are these? Are they mosquitoes or other Dipterans? It should be indicated at the very least what Insect Order they belong to since the information being presented here is comparative.

**Response 23.** *Aenasius bambawalei* belong to Hymenoptera order of insects. Whereas belong to order Coleoptera. These have been compared as the number of OBPs and ORs are highly variable.

**Point 24.** Line 304-305. “which are less than the other two chromosomes in An. gambiae”.
Why is An. gambiae mentioned here. Shouldn’t this be referencing the other two chromosomes in An. stephensi?

**Response 24.** Thanks for pointing out. It has been corrected in the manuscript.

**Point 25.** Line 308-309. “Like OBPs, ORs are also concentrated on chromosome 3.”
This statement is logically inconsistent. On Line 303, it is mentioned that most of the OBP genes are localized on chromosome 2, not chromosome 3. So this is not a correct “like…also” statement of logic, since the patterns being compared are different. Consider to re-word this.

**Response 25.** This sentence has been corrected in the manuscript as well.

**Point 26.** Line 318-319. “Likewise, Culex quinquefasciatus OBPs and ORs showed the closer relationship with OBPs and ORs of Ae. aegypti”
Closer compared to what? What exactly is the reference point? An. gambiae? D. melanogaster? Some other species?

**Response 26.** Reference point in this case is the closer relationship of OBPs and ORs with other closely related organisms as compared to the distantly related organisms. As *Culex quinquefasciatus* OBPs and ORs are closely related to those of *Aedes aegpti* as compared to OBPs and ORs of *D. melanogaster*.

**Point 27.** Line 328/329. Again here with the “significant PGP domain…insignificant PGP-OBP domain”.
Significance is usually indicated to refer to statistical significance. So it is not clear what is meant by significance versus insignificance in this sense.

**Response 27**. Significant and insignificant are the terms used in the Pfam database. Insignificant indicates that these sequences had insignificant match to the Pfam PBP/GOBP domain family. Plus-C OBPs have extra conserved two cysteines and one proline residues, therefore, Pfam indicated the insignificant match with PBP/GOBP domain family. But these sequences were confirmed as OBPs using multiple sequence alignment having clear Plus-C OBPs motif pattern.

**Point 28.** Line 333-334, Line 335-336. Here it says “average molecular weight of the OBPs is 18 kDA” first time and then “Average molecular weight of the OBPs is also reported to be around 50 kDA the second time.
Cannot be correct for both as written. Do you mean to say ORs for the second instance?

**Response 28.** Thanks for pointing out. Yes, it was meant to be 50 kDA for ORs. This has been corrected in the manuscript.

**Point 29.** Figure 6, in-figure legend it says OBPs, but this figure is depicting phylogeny of the ORs. This needs to be corrected.

**Response 29.** Yes. It has been corrected. As figure has been revised, so this has been corrected as well.

**Point 30.** Figure 9B. In the in-figure legend it shows a PFAM domain indicated as PBP/GOBP. However, the PBP/GOBP subfamily of OBPs is known to be specific only to Lepidopterans, however this report is focused on a Dipteran mosquito. So it is not clear how a PBP/GOBP domain would be relevant to OBPs of this species. It is cautioned against relying too much on PFAM domains due to inaccuracies such as this. It gives the impression that all of these proteins are members of the PBP/GOBP subfamily, which they are not.

**Response 30.** This domain represents the General Odorant Binding Proteins (GOBPs). All the OBPs and ORs sequences of *Ae. aegypti*, *An. gambiae*, and *D. melanogaster* were checked for the presence of domain. They all contained PBP/GOBP domain. However, conserved amino acid pattern was also assessed by multiple sequence alignment.
