# Supplementary material for: Odorant Binding Proteins (OBPs) and Odorant Receptors (ORs) of Anopheles stephensi: Identification and comparative insights
Source: PLoS One. 2022 Mar 22;17(3):e0265896. doi: 10.1371/journal.pone.0265896 (PMC8939812; doi:10.1371/journal.pone.0265896)
Supplement: Attachment — Submitted filename: Response to Reviewer 2.docx [file pone.0265896.s008.docx]

**Response to Reviewer 2**

**Point 1.** In Figures 5 and 6, the authors have added bootstrap values. In the figure legends for these figures, it should be indicated that these numbers represent bootstrap values. In these figures, some of the sequence IDs are different colors, including those that are in black font. The representations of the different colors should also be indicated in the appropriate figure legends. Additionally, the presentation of the bootstrap values in these figures is somewhat disorganized, sometimes with the numbers being overlapping and obscured by the phylogenetic relationship lines. This should be avoided, and numbers should be positioned such that they are not overlapping with other elements of the figure and it should be clear which phylogenetic relationships they are referring to.

**Response 1.** Thanks for pointing it out. The position of the values has been corrected and legends have been modified in the manuscript as well.

**Point 2.** In Figures 7 and 8, it is not clear to the reader what the grey synteny lines (figures 7 and 8) represent relative to the green (figure 7), blue (figure 7) or red ones (figure 8). These representations should be clarified in the appropriate figure legends

**Response 2.** Description of these lines has been provided. These lines show the syntenic relationship between two homologous genes in the compared organisms in the figures.

**Point 3.** It was previously requested for the authors to update the tables to include ORF completion status for each OR/OBP as well as the size of the ORF, in terms of number of amino acids. This information is typically standard to include in these kinds of reports, though it has not been provided here yet. It is again requested to provide this information, either as an expansion of tables 1 and 2, appended to Figures 9 and 10, or else included as supplementary material.

**Response 3.** Thanks for highlighting this point. This information has now been included in the supplementary material (Supplementary Table 1 and Supplementary Table 2).

**Point 4.** On lines 75,76, it is mentioned that “ORs have been previously identified based on in situ hybridization using RNA probes and transcriptomic data from different organisms”. However, it is also the case that ORs have been previously identified through analysis of genomic data, as with An. gambiae and numerous other species. This approach should also be mentioned here.

**Response 4.** Yes, ORs have been identified by analyzing the genomic data and this has been added in the manuscript as per your kind suggestion.

**Point 5.** In the first paragraph of the Discussion, from Lines 308 to 323, total number of OBPs and ORs are presented. Number of OBPs are compared to total numbers from An. gambiae, Ae. aegypti, and D. melanogaster (Lines 310-311), however a similar comparison of ORs between An. stephensi and these three species is not included towards the end of this paragraph. It would seem relevant to also compare total number of ORs across these four species.

**Response 5.** Thanks for this important suggestion. This information has been updated in the manuscript.

**Point 6.** On Line 377-378, “Molecular simulations helps in the retention of ligand-receptor interactions for an extended period to induce behavioral response, thus helping to identify novel attractants.” This statement is unclear. It is unclear what is meant how molecular simulations help in the retention of ligand-receptor interactions? Do the authors mean to say that the molecular simulations help to identify/predict extended ligand-receptor interactions that are retained sufficiently long enough to possibly induce behavioral response? Or is something else meant? As written it is not clear how simulations could help in retention of anything.

**Response 6.**  Molecular simulation helps to check the retention of protein and ligand bonds for a specified duration to analyze their bonding. This is widely used in drug discovery as well as in the prediction of novel repellents for insects using the OBPs. So, the identification of the *Anopheles stephensi* OBPs will help in the identification of novel repellents as well as attractants by means of molecular docking and molecular dynamics simulations.
