# Supplementary material for: Odorant Binding Proteins (OBPs) and Odorant Receptors (ORs) of Anopheles stephensi: Identification and comparative insights
Source: PLoS One. 2022 Mar 22;17(3):e0265896. doi: 10.1371/journal.pone.0265896 (PMC8939812; doi:10.1371/journal.pone.0265896)
Supplement: Attachment — Submitted filename: Response to Reviewer 2.docx [file pone.0265896.s009.docx]

Response to Reviewer 2

Point 1. One is a typo found on line 272 of the untracked version. There should be a space in between "homology" and "with". In present version it is written as one word.

Response 1. Thanks for pointing it out. This typo has been corrected in the manuscript.

Point 2. More substantially, in the new Supplementary Tables, specifically the Table S2 with the ORs, the complete/incomplete status for the ORs seems not to be filled in (as was the case for OBPs in Table S1), for column 3. This is critical information to include because it appears several of the ORs are incomplete. Also in the figure legend for Table S2, it is written that "Similarly, OBP's names and accessions have also be given". This should be changed to indicate that it is the OR's names.

Response 2. The complete and incomplete status of ORs has been updated in the table. You have pointed it right as some ORs have incomplete ORFs that have been updated in the manuscript. The caption of Table S2 has been corrected as per your kind suggestion.
